# Persistent barrage firing in cortical interneurons can be induced *in vivo* and may be important for the suppression of epileptiform activity

**DOI:** 10.3389/fncel.2014.00076

**Published:** 2014-03-13

**Authors:** Norimitsu Suzuki, Clara S.-M. Tang, John M. Bekkers

**Affiliations:** Eccles Institute of Neuroscience, The John Curtin School of Medical Research, The Australian National University, CanberraACT, Australia

**Keywords:** action potential, inhibition, interneuron, neurogliaform, patch clamp, piriform cortex, seizure, synapse

## Abstract

Neural circuits are typically maintained in a state of dynamic equilibrium by balanced synaptic excitation and inhibition. However, brain regions that are particularly susceptible to epilepsy may have evolved additional specialized mechanisms for inhibiting over-excitation. Here we identify one such possible mechanism in the cerebral cortex and hippocampus of mice. Recently it was reported that some types of GABAergic interneurons can slowly integrate excitatory inputs until eventually they fire persistently in the absence of the original stimulus. This property, called persistent firing or retroaxonal barrage firing (BF), is of unknown physiological importance. We show that two common types of interneurons in cortical regions, neurogliaform (NG) cells and fast-spiking (FS) cells, are unique in exhibiting BF in acute slices (~85 and ~23% success rate for induction, respectively). BF can also be induced *in vivo*, although the success rate for induction is lower (~60% in NG cells). In slices, BF could reliably be triggered by trains of excitatory synaptic input, as well as by exposure to proconvulsant bath solutions (elevated extracellular K^+^, blockade of GABA_A_ receptors). Using pair recordings in slices, we confirmed that barrage-firing NG cells can produce synaptic inhibition of nearby pyramidal neurons, and that this inhibition outlasts the original excitation. The ubiquity of NG and FS cells, together with their ability to fire persistently following excessive excitation, suggests that these interneurons may function as cortical sentinels, imposing an activity-dependent brake on undesirable neuronal hyperexcitability.

## INTRODUCTION

The computational power of cortical microcircuits is thought to be enhanced by the presence of recurrent excitatory connections that can selectively amplify weak inputs. However, this type of architecture is potentially dangerous because over-excitation may lead to seizures (Shepherd, 2011). To counter this tendency, the cortex contains various types of inhibitory circuits that maintain the network in a state of dynamic equilibrium. Here we explore the idea that a recently described feature of some types of GABAergic interneurons, called persistent firing (Sheffield et al., 2011) or retroaxonal barrage firing (BF; Sheffield et al., 2013), may equip those interneurons with an additional mechanism for protecting against cortical over-excitation.

Persistent or retroaxonal barrage firing (which we will call “BF”) is an unusual kind of slow integration of synaptic excitation in which hundreds of action potentials (APs), evoked over several seconds, eventually initiate spontaneous AP firing that sometimes lasts for minutes (Sheffield et al., 2011). BF is different from other forms of persistent firing (Egorov et al., 2002; Major and Tank, 2004; Fransen et al., 2006; Thuault et al., 2013) because it is restricted to inhibitory interneurons and the APs appear to be initiated in the distal axon. The induction mechanism for BF is unclear, but it might involve accumulation of extracellular K^+^, changes in Ca^2^^+^ signaling, or intercellular communication via gap junctions (Sheffield et al., 2011, 2013). BF has been reported to occur in a subclass of interneurons of the hippocampus and neocortex (Sheffield et al., 2011, 2013) and in neurogliaform (NG) and fast-spiking (FS) interneurons of the hippocampus (Krook-Magnuson et al., 2011). However, none of these studies directly tested hypotheses about the possible functions of this type of activity.

In this paper we examine the possibility that BF is a mechanism by which excess excitation initiates compensatory feedback inhibition that outlasts the original excitation. By prolonging and generalizing synaptic inhibition, BF could function as an endogenous anticonvulsant mechanism. We show that this form of firing has a number of characteristics that are compatible with such a role.

## MATERIALS AND METHODS

### ETHICAL APPROVAL

All animal housing, breeding and surgical procedures were approved by the Animal Experimentation Ethics Committee of the Australian National University and conform to the guidelines of the National Health and Medical Research Council of Australia.

### ANIMALS

The majority of experiments used heterozygous GAD67-GFP (Δneo) mice of either sex in which green fluorescent protein (GFP) is specifically expressed in neurons containing γ-aminobutyric acid (GABA; Tamamaki et al., 2003; Suzuki and Bekkers, 2010b). Here we use the shorthand GAD67-GFP when referring to these animals. GAD67-GFP mice are bred on a C57BL6/J background and have normal behavior and neuroanatomy (Kerlin et al., 2010; Suzuki and Bekkers, 2010b; Zhan and Luo, 2010). GFP^+^ cells in these mice have the electrical and morphological features of normal GABAergic interneurons (Suzuki and Bekkers, 2010a). Use of GAD67-GFP mice greatly facilitated targeted recordings from inhibitory interneurons. However, some experiments used wildtype C57BL6/J mice and yielded identical results.

### SLICE PREPARATION

Experiments used acute brain slices (300 μm thick) prepared from 59 GAD67-GFP or wildtype C57BL6/J mice (18–45 d-old). Standard methods of slice preparation were used (Suzuki and Bekkers, 2010a, 2011, 2012). Briefly, mice were deeply anesthetized with isoflurane (2% in oxygen) then rapidly decapitated. Using a vibrating slicer (Campden Instruments), coronal slices of the cortex or transverse slices of the hippocampus were prepared under ice–cold cutting solution containing (in mM) 125 NaCl, 3 KCl, 0.5 CaCl_2_, 6 MgCl_2_, 25 NaHCO_3_, 1.25 NaH_2_PO_4_, and 10 glucose (osmolarity 305 mOs/kg), bubbled with 5% CO_2_/95% O_2_ (carbogen). The slices were incubated for 1 h at 35°C in a holding chamber containing carbogen-bubbled artificial cerebrospinal fluid (ACSF; composition below), then were held at room temperature until required.

### SURGERY FOR *IN VIVO* EXPERIMENTS

These experiments used 17 GAD67-GFP mice (30–45 d-old). Animals were anesthetized with chlorprothixene (5 mg/kg I.P.) and urethane (0.5–1 g/kg I.P.). When the pinch reflex was completely absent (typically after 10–20 min), animals were placed on a heating blanket to maintain physiological body temperature. An injection of a local anesthetic (prilocaine hydrochloride, 0.2 mg/ml S.C.) was delivered to the scalp, then the skin over the top of the head was retracted and a craniotomy performed to expose a small region (~3 mm diameter) of the primary somatosensory cortex. The *dura mater* was left intact. Depth of anesthesia was monitored frequently using the pinch reflex test. A top-up dose of urethane (half the initial dose) was typically required 4–6 h after the start of surgery. Saline (100–200 μl I.P.) was administered every hour to maintain hydration. At the end of the experiment the animal was killed by an overdose of urethane and decapitation without being allowed to regain consciousness.

### SLICE ELECTROPHYSIOLOGY

Infrared-differential interference contrast videomicroscopy was used to make visualized whole-cell patch clamp recordings from GFP^+^ neurons in anterior piriform cortex, primary somatosensory cortex and area CA1 of the hippocampus, or from GFP^-^ glutamatergic principal neurons in layer 2 of the piriform cortex. The different classes of piriform cortex GABAergic interneurons were identified by their characteristic electrical properties, morphologies and laminar location, as previously described (Suzuki and Bekkers, 2010a,b). NG cells were initially identified by their very bright GFP fluorescence, and FS cells by their larger and more weakly fluorescent somata located in layer 3. The identities of both cell types were subsequently confirmed by their intrinsic electrical properties and morphologies (Suzuki and Bekkers, 2010a).

Slices were continuously superfused (2–3 ml/min) with ACSF containing (mM) 125 NaCl, 3 KCl, 2 CaCl_2_, 1 MgCl_2_, 25 NaHCO_3_, 1.25 NaH_2_PO_4_, and 25 glucose (310 mOs/kg), bubbled with 5% CO_2_/95% O_2_ (carbogen) and maintained at 33–35°C. In some experiments (**Figure 5**) the concentration of KCl in the ACSF was raised to 7 mM or 9 mM. For current clamp recordings, patch electrodes had resistances of 6–10 MΩ when filled with internal solution containing (mM) 135 KMeSO_4_, 7 NaCl, 0.1 EGTA, 2 Na_2_ATP, 2 MgCl_2_, 0.3 GTP, 10 HEPES at pH 7.2, supplemented with 0.2–0.4% biocytin (295–300 mOs/kg). This solution had a Cl^-^ concentration of 11 mM and a measured junction potential of -7 mV. For voltage clamp recordings (e.g., **Figure 6**), 135 mM CsCl replaced the KMeSO_4_ (giving a junction potential of 0 mV) and electrodes had resistances of 5–7 MΩ. The presence of Cs in this internal solution blocked the K channels that mediate the GABA_B_ response. The holding potential was -90 mV. All voltages given in this paper are corrected for junction potentials. Unless stated otherwise, all compounds were obtained from Sigma-Aldrich.

Neurons were visualized using a BX51WI microscope with a 40×/0.8 NA objective (Olympus). Electrical data were acquired using a Multiclamp 700A amplifier (Molecular Devices). For current clamp recordings, the cell was allowed to remain at its resting membrane potential. Bridge balance and capacitance neutralization were carefully adjusted and checked for stability. For voltage clamp recordings, series resistance was monitored for stability but series resistance compensation was not used. Voltage or current traces were filtered at 10 kHz and digitized at 20 or 50 kHz by an ITC-18 interface (Instrutech/HEKA) under the control of Axograph (Axograph Scientific).

Focal extracellular synaptic stimulation (e.g., **Figure 5A**) was done using a custom-built isolated stimulator that delivered a 100 μs-long constant current pulse with an adjustable amplitude. The stimulating electrode was constructed from a patch electrode (tip diameter ~5 μm) filled with 1 M NaCl and coated with electrically conductive paint. The stimulus current was passed between the filling solution and a wire connected to the paint; hence, this functioned as a concentric bipolar stimulating electrode (Bekkers and Clements, 1999). The tip of the stimulating electrode was placed 200 μm from the soma of the recorded cell, in the same layer, to avoid directly stimulating the axon of the cell.

**FIGURE 1:**
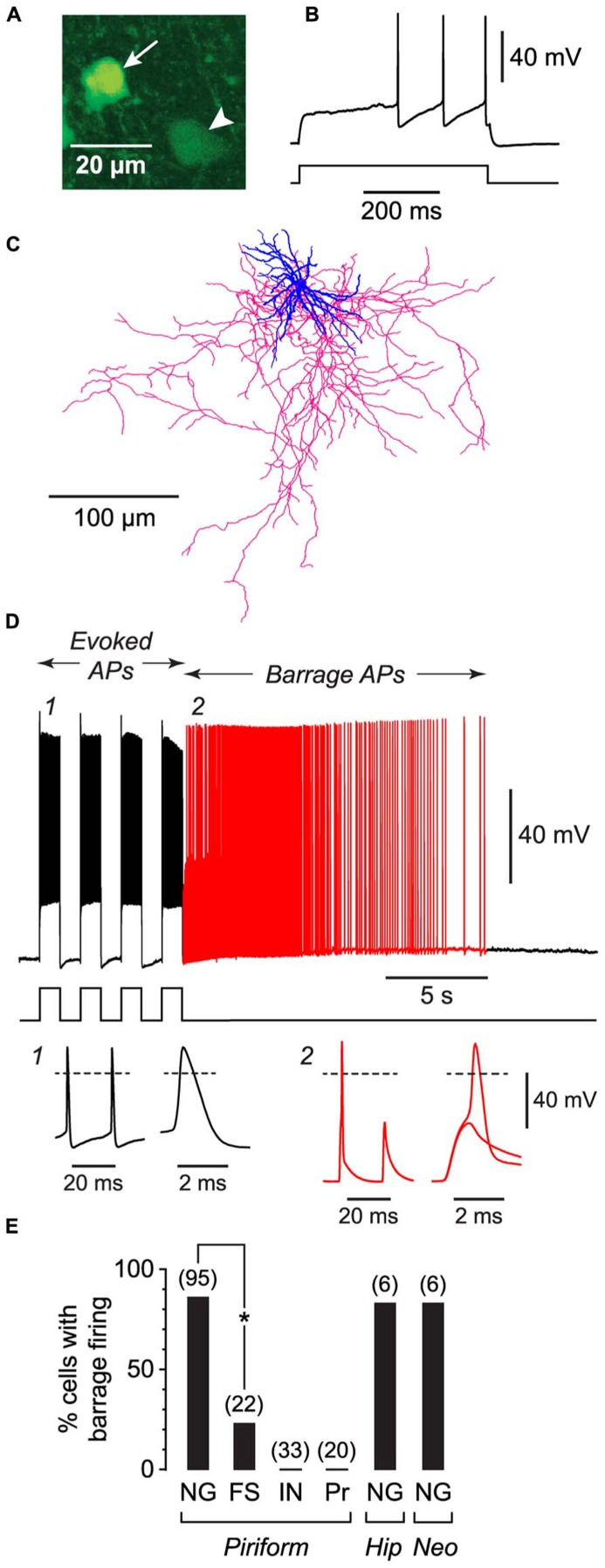
**NG cells preferentially exhibit persistent barrage firing.** All data shown in this figure were obtained from acute brain slices. **(A)**, Fluorescence image of a region in layer 3 of the piriform cortex from a GAD67-GFP mouse, showing the bright GFP fluorescence in the soma of a NG cell (arrow) compared with the weak GFP fluorescence in a nearby non-NG cell (arrowhead). **(B)**, Response of the arrowed NG cell in **(A)** to a depolarizing current step (220 pA) just above rheobase. Note the delay to spiking, characteristic of NG cells. **(C)**, Tracing of the same cell as in **(A)**, showing the dense arbor of dendrites (blue) and axons (red) typical of NG cells (Suzuki and Bekkers, 2010a). **(D)**, Induction of BF in the same cell as in **(A)**. Induction comprised a series of depolarizing 800 pA current steps (shown below) that were ceased when BF commenced (red part of trace). Insets, bottom, show action potentials at the numbered locations in the main panel, displayed on two different time scales. Note the spikelet (small amplitude AP) in inset 2. The spikelet and full-height spike in this inset are shown superimposed on an expanded timebase on the right, showing that the full-height spike appears to ride on the back of a spikelet. Dashed lines, 0 mV. **(E)**, Percentage of cells showing BF for different types of neurons. FS, fast-spiking; IN, other interneuron types in the piriform cortex (regular-spiking, horizontal, bitufted); Pr, layer 2 principal cells; Hip, hippocampus (CA1 *Stratum lacunosum-moleculare*); Neo, somatosensory neocortex (layers 1–3). Number of induction attempts is given in parentheses. Asterisk, significantly different (*p* = 2 × 10^-6^, Fisher’s exact test).

**FIGURE 2:**
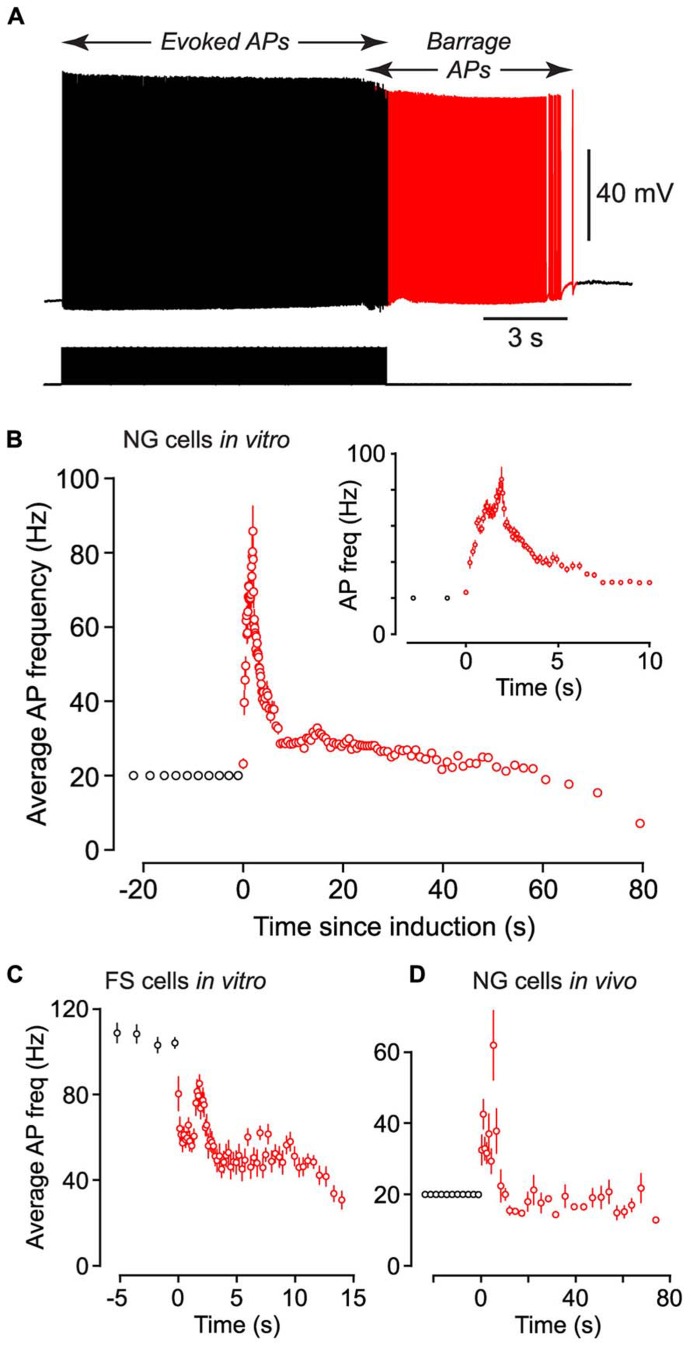
**Following induction of BF (both *in vivo* and *in vitro*) the average firing frequency of persistent APs shows characteristic changes.** **(A)**, Example of raw data from a NG cell in an acute brain slice, showing BF induced by a 20 Hz train of depolarizing current steps (each 1.4 nA for 2 ms; stimulus pattern at bottom). Red portion of trace indicates period of BF. **(B)**, Average frequency of APs measured in *n* = 27 NG cells plotted against time since induction of BF. Induction used a 20 Hz train of depolarizing current steps (1–1.5 nA for 2 ms). Black and red symbols represent average AP frequency during the induction and BF periods, respectively. Error bars indicate ±SE. Note that the time for induction of BF varied between ~8 and ~38 s in different cells. Inset shows part of the same plot expanded horizontally. **(C)**, A similar plot of average AP frequency against time for *n* = 3 FS cells recorded in acute slices of the piriform cortex. BF in these experiments was triggered by 1 s-long depolarizing current steps repeated at 1 Hz. **(D)**, A similar plot of AP frequency versus time for *n* = 12 NG cells recorded *in vivo* from the somatosensory cortex of anesthetized mice, following induction by a 20 Hz train of 2 ms-long depolarizing current steps.

**FIGURE 3:**
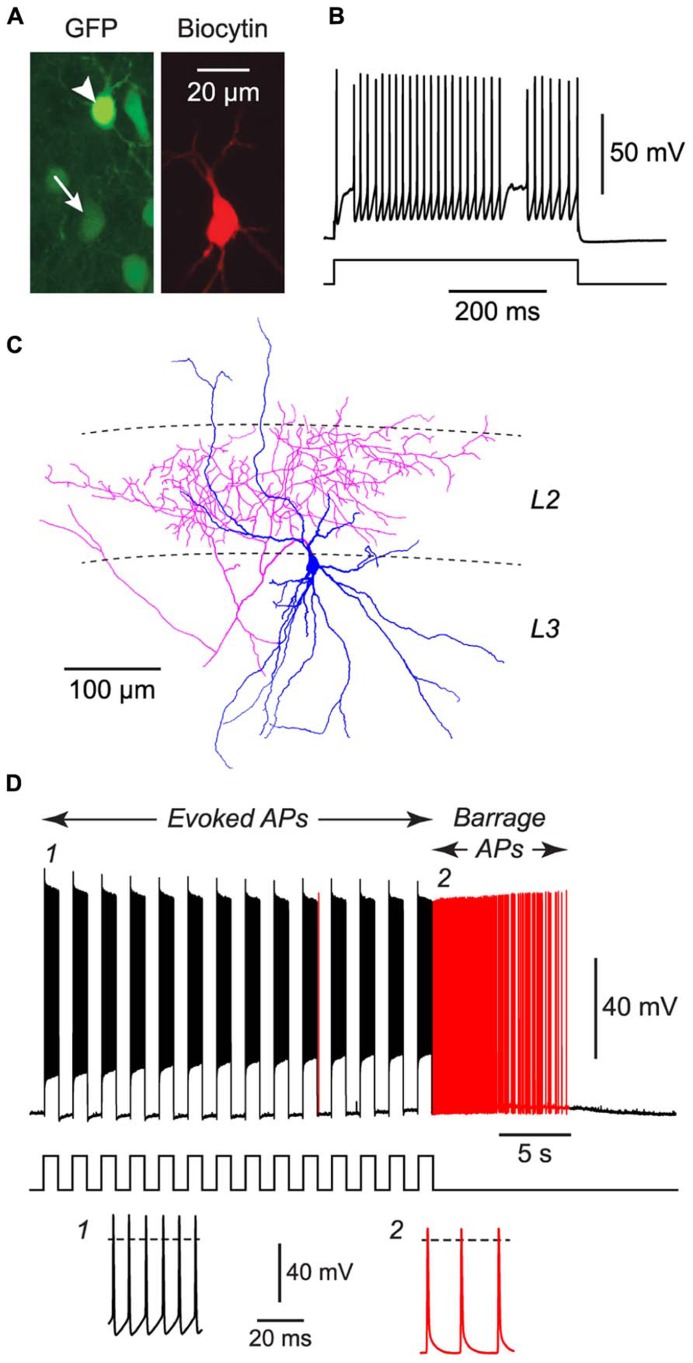
**FS cells in acute slices of the piriform cortex can also exhibit BF.** **(A)**, Fluorescence images of the same region of a slice of piriform cortex, showing GFP label in this GAD67-GFP mouse (left) and the biocytin fill of the neuron from which recordings were made (middle). The recorded FS cell has weak GFP fluorescence (arrow, bottom), contrasting with strong GFP fluorescence in a nearby NG cell (arrowhead, top). **(B)**, Response of the filled FS cell in **(A)** to a current step (680 pA), showing its fast-spiking phenotype. **(C)**, Tracing of the same cell as in **(A)**, showing the multipolar dendrites (blue) and dense axonal projections (red) to layer 2 (*L2*) that are typical of FS cells in the piriform cortex (Suzuki and Bekkers, 2010a). **(D)**, Response of the cell in panels **(A–C)** to a series of current steps (1 nA for 1 s, repeated at 0.5 Hz) that eventually elicited BF (red portion of trace). Stimulus pattern is shown at bottom. Insets, bottom, show action potentials at the numbered locations in the main panel. Dashed lines, 0 mV.

Barrage firing was triggered using three types of electrical stimuli: (i) 1 s-long depolarizing current steps (0.5–1.5 nA) applied via the somatic electrode at 0.5 Hz; each step elicited trains of 20–60 APs in NG cells (e.g., **Figure 1D**) or 100–160 APs in FS cells (e.g., **Figure 3D**). (ii) 2 ms-long depolarizing current steps (1.0–1.5 nA) applied at the soma at 20 Hz; each step elicited a single AP (e.g., **Figure 2A**). (iii) A train of extracellular synaptic stimuli applied at 20 Hz; each stimulus elicited an excitatory postsynaptic potential that was suprathreshold for firing a single AP (e.g., **Figure 5A**). Shortly after BF emerged, the stimulus was stopped and data acquisition continued for as long as the spontaneous firing lasted. If BF did not appear after 80 s of continuous stimulation, the stimulus was stopped for 2 min, then another train of up to 80 s duration was applied. If BF did not appear after three such trains, the cell was noted as being unable to generate this form of firing. In some experiments, BF was triggered by perfusing the bath with modified ACSF (**Figures 5B,C**).

At the conclusion of the experiment, the patch electrode was carefully retracted while maintaining the seal. The slice was fixed for 1 h in 4% paraformaldehyde in phosphate buffer then stored in phosphate buffer at 4°C until processing. Fluorescence images (e.g., **Figure 1A**) were obtained using a Zeiss Pascal confocal microscope with a 20×/0.75 NA objective. Image projections were made from a series of 4–10 frames at 5 μm intervals.

### *IN VIVO* ELECTROPHYSIOLOGY

A two-photon microscope (Sutter MOM) was used to make targeted whole-cell patch clamp recordings from identified GFP^+^ interneurons in the upper ~200 μm (layers 1 and 2/3) of the primary somatosensory cortex of mice. Imaging was performed using a Chameleon Ultra Ti:Sapphire laser (Coherent) operating at 800 nm and a 40×/0.8 NA objective (Olympus). The mouse was placed on an electrically heated blanket to maintain a physiological core temperature, and its head was secured via a metal rod attached to the skull with dental cement. A small chamber was constructed around the craniotomy and filled with saline. The reference electrode was a chlorided silver wire inserted under the skin of the neck.

**FIGURE 4:**
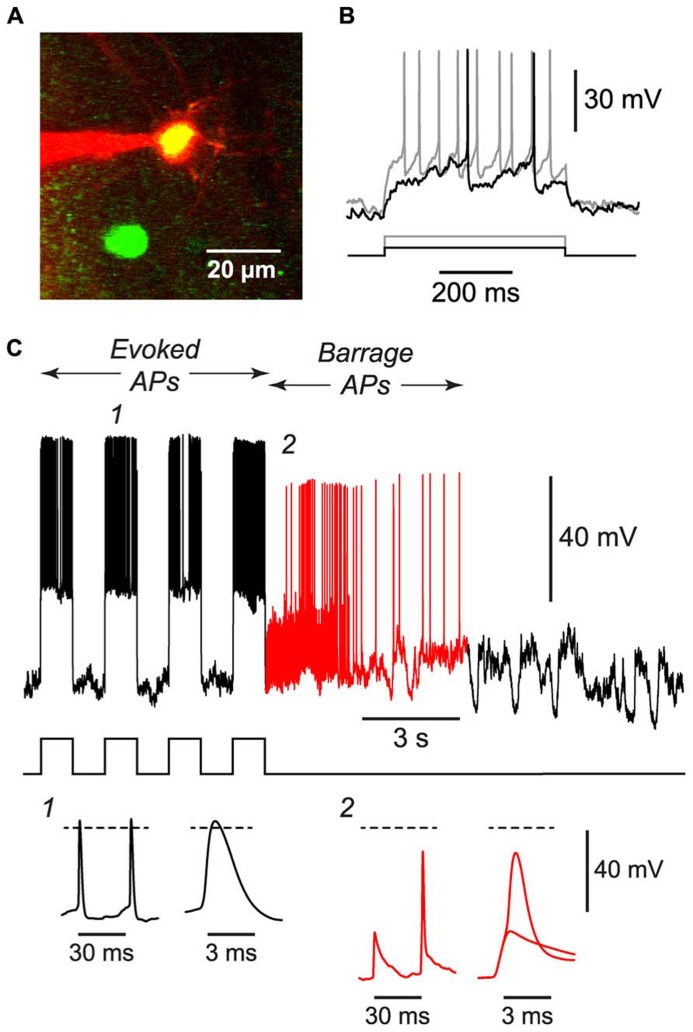
**BF can be induced in NG cells *in vivo*.** **(A)**, 2-photon microscope image showing a whole-cell patch electrode (red) on a NG cell in upper layer 2 of the somatosensory cortex of an anesthetized GAD67-GFP mouse. This image is a merge of two z-projections, one (green) showing GFP, the other (red) showing the Alexa 594 dye in the solution in the patch electrode. **(B)**, Responses of the cell in **(A)** to depolarizing current steps (black, 50 pA; gray, 120 pA) showing the delayed firing and lack of accommodation typical of NG cells. **(C)**, Example of the BF induced by a train of current steps (240 pA for 1 s; stimulus pattern at bottom) in the NG cell shown in panels **(A,B)**. Red portion of trace indicates period of BF. Insets, bottom, show action potentials at the numbered locations in the main panel, displayed on two different time scales. The spikelet and full-height spike in inset 2 are shown superimposed on an expanded timebase on the right. Dashed lines, 0 mV. Elevated electrode resistance attenuated the AP amplitudes in this experiment, and difficulties in adjusting the bridge balance accounts for the apparent larger peak amplitude of evoked APs (see Materials and Methods).

Patch electrodes and the internal solution were the same as those used in the slice current clamp experiments, except that the internal solution also contained Alexa 594 (20 μM, Invitrogen) to enable visualization of the electrode in the red channel (**Figure 4A**). Putative NG cells were identified by their very bright fluorescence in the green channel (**Figure 4A**). A patch electrode was advanced through the *dura mater* while applying high pressure (25 kPa) to the back of the electrode, then the pressure was reduced (6–8 kPa) and a targeted whole-cell recording was obtained in the usual way. Electrode series resistance was higher than is typical in the acute slice preparation; hence, the bridge balance was difficult to adjust accurately, and APs were often attenuated and sometimes did not overshoot 0 mV (e.g., **Figure 4C**). However, by all other criteria (e.g., resting membrane potential, input resistance, cell morphology) the neurons were healthy. BF was induced by a series of 1 s-long depolarizing current steps (200–500 pA) applied via the patch electrode at 0.5 Hz. At the end of the experiment an image stack was acquired in the red channel (10 μm interval) to document the dendritic arbor of the cell. In some experiments the animal was perfused with 4% paraformaldehyde and the morphology of the cell was recovered.

### ANALYSIS

Neuronal morphology (e.g., **Figure 1C**) was revealed using an ABC kit (Vector Laboratories) and diaminobenzidine, or by labeling with streptavidin-Alexa 594 (Invitrogen; e.g., **Figure 3A**). Cell tracing was done manually using the Neurolucida tracing system (MBF Bioscience). All electrophysiological analysis was done using either Axograph or Igor Pro (Wavemetrics). For the “1 s steps” induction protocol (1 s-long current steps repeated at 0.5 Hz; e.g., **Figure 1D**; **Table 1**), average AP frequency during induction was calculated only for the periods of current injection. When measuring the instantaneous AP frequency with respect to BF onset (**Figures 2B–D**), any stimulus-evoked APs occurring after onset were disregarded. Spikelets as well as full-height APs were counted for this analysis. The plots in **Figures 2B–D** were obtained by combining instantaneous frequency data from all cells then averaging over successive time windows, whereas the average peak frequencies given in **Table 1** were found by averaging the individual peak frequencies found for each cell. The mean voltage threshold, rise time and peak of APs during induction (“Induct”) and of barrage-firing APs (“BF”), given in **Table 1**, were calculated either by averaging five consecutive APs near the start of induction (for “Induct” data) or by averaging the first five spontaneous APs after the start of BF (for “BF” data). AP voltage threshold was measured as the membrane potential (*V_m_*) at which *dV_m_/dt* first exceeded 50 mVms^-1^. AP risetime was the time between the voltage threshold and the peak of the AP. All errors represent ± standard error (SE), with *n* the number of cells. Statistical tests used Fisher’s exact test or ANOVA, as indicated, or the paired or unpaired *t*-test if not explicitly stated.

**Table 1:** Summary of parameters associated with barrage firing in different neuron types, brain regions and recording conditions.

	*% Cells with BF*	*% BF cells with spikelets*	*Number of APs before BF starts*	*AP freq during induction (Hz)*	*Max BF frequency (Hz)*	*BF duration (s)*	AP threshold (mV)		AP rise time (ms)	
							*Induct*	*BF*	*Induct*	*BF*
Piriform NG cells *in vitro*	85% (60)	69% (51)	437 ± 27 (50)	49.7 ± 2.9 (47)	104.0 ± 12.3 (46)	35.6 ± 4.7 (49)	-41.0 ± 1.0 (47)	-77.0 ± 0.9 (47)	0.39 ± 0.01 (47)	1.00 ± 0.04 (47)
Piriform FS cells *in vitro*	23% (22)	20% (5)	1136 ± 188 (5)	128.7 ± 29.9 (5)	119.5 ± 10.7 (4)	9.8 ± 2.3 (5)	-48.0 ± 1.4 (5)	-75.5 ± 2.2 (5)	0.26 ± 0.01 (5)	0.46 ± 0.05 (5)
CA1 NG cells *in vitro*	83% (6)	20% (5)	370 ± 71 (5)	41.4 ± 3.7 (4)	62.6 ± 14.3 (5)	47.6 ± 26.3 (5)	-48.9 ± 2.2 (4)	-76.4 ± 2.0 (4)	0.37 ± 0.02 (4)	0.56 ± 0.04 (4)
Neocortical NG cells *in vitro*	83% (6)	60% (5)	391 ± 70 (5)	30.7 ± 2.4 (5)	69.7 ± 12.7 (4)	29.7 ± 16.0 (4)	-41.9 ± 4.4 (4)	-76.2 ± 5.0 (4)	0.35 ± 0.01 (4)	0.72 ± 0.09 (4)
Neocortical NG cells *in vivo*, 1 s and 2 ms step induct	61% (33)	45% (20)	851 ± 96 (20)	27.0 ± 2.3 (20)	99.5 ± 19.1 (18)	35.7 ± 10.9 (20)	-32.1 ± 2.7 (14)	-66.4 ± 2.2 (14)	0.63 ± 0.05 (14)	1.14 ± 0.11 (14)
Piriform NG cells *in vitro*, EPSP induct	80% (5)	0% (4)	272 ± 97 (4)	19.1 ± 0.5 (4)	95.0 ± 12.8 (4)	33.2 ± 22.3 (4)	-47.2 ± 3.4 (4)	-72.9 ± 1.7 (4)	0.39 ± 0.02 (4)	0.71 ± 0.04 (4)
Piriform NG cells *in vitro*, K^+^ induct	63% (8)	0% (5)	–	–	119.0 ± 13.2 (5)	–	-49.0 ± 2.1 (3)	-57.8 ± 0.8 (3)	0.41 ± 0.05 (3)	0.55 ± 0.05 (3)

*All *in vitro* data shown here were obtained from slices prepared from 18 to 25 d-old mice, whereas *in vivo* data were from 30 to 45 d-old mice. Format of entries is: mean ± SE (number of cells). “Max BF freq (Hz)” was calculated by combining spikelets and composite (full-amplitude) barrage-firing APs. “AP rise time (ms)” was measured only for composite barrage-firing APs. In the right-most four columns, “*Induct*” indicates parameters averaged for five APs near the start of induction, “*BF*” indicates parameters averaged for the first five APs after start of barrage firing. Unless otherwise stated, induction used 1 s-long current steps at 0.5 Hz. See Results for illustrative statistical comparisons.*

## RESULTS

### ONLY TWO CLASSES OF INTERNEURONS IN THE PIRIFORM CORTEX EXHIBIT BARRAGE FIRING

We began by making whole-cell recordings from neurons in acute slices from the anterior piriform cortex, which is known to be highly epileptogenic (Piredda and Gale, 1985). For these initial experiments, slices were prepared from young (18–25 d-old) mice. We took advantage of our earlier work classifying the different subtypes of GABAergic interneurons in the piriform cortex (Suzuki and Bekkers, 2010a) to record from specific classes of interneurons. For example, NG interneurons were identified by their compact dendritic morphology (Price et al., 2005; **Figure 1C**), very bright expression of GFP in tissue from GAD67-GFP mice (Suzuki and Bekkers, 2010a; **Figure 1A**), and distinctive delay to firing close to threshold (Capogna and Pearce, 2011; **Figure 1B**). For each neuron we attempted to induce BF by applying depolarizing current steps to elicit trains of evoked APs. Two types of current step patterns were used: 1 s-long steps at 0.5 Hz (**Figure 1D**) and 2 ms-long steps at 20 Hz (**Figure 2A**). BF, when present, typically emerged after 5–20 s of stimulation (e.g., **Figure 1D**). We scored cells as being unable to generate this form of firing if it did not emerge after three trains of stimulation, each lasting 80 s.

Barrage firing was very common in NG cells in all layers of the piriform cortex (86% of cells with both induction protocols; *n* = 95; **Figure 1E**). Using the 1 s step induction protocol, BF required 437 ± 27 evoked APs for its emergence, and it lasted 35.6 ± 4.7 s (mean ± SE, *n* = 47–49; **Table 1**). Similar results were obtained for the 2 ms step induction protocol (452 ± 38 APs; 27.0 ± 5.9 s duration; *n* = 28; not significantly different from 1 s step induction, *p* > 0.7). In agreement with previous work (Sheffield et al., 2011, 2013), barrage-firing APs had a dramatically hyperpolarized voltage threshold (significantly different from regular APs, *p* < 0.01, ANOVA; **Table 1**; **Figure 1D**, insets 1, 2). Isolated barrage-firing spikelets were frequently seen (**Figure 1D**, inset 2), occurring in 69% of NG cells that expressed this kind of firing (**Table 1**). Full-height barrage-firing APs appeared to comprise an initial spikelet upon which rode an AP of normal amplitude (**Figure 1D**, inset 2 with expanded time scale). The resulting inflection in the rising phase of full-height barrage-firing APs gave them a significantly slower rise time compared with normal APs (*p* < 0.01, ANOVA; **Table 1**).

All of these properties are consistent with barrage spike initiation in the distal axon (Sheffield et al., 2011, 2013). It is thought that barrage-firing spikes propagate retroaxonally toward the soma, appearing there as attenuated spikelets that rise from a strongly hyperpolarized voltage threshold (**Figure 1D**, inset 2). A spikelet may or may not trigger a full-amplitude perisomatic spike (**Figure 1D**, inset 2). That is, the critical feature of a barrage-firing spike is its hyperpolarized voltage threshold, not its amplitude, which might be small (for a spikelet alone) or large (for a composite AP made up of a spikelet and a perisomatic spike). Unless otherwise stated (legend to **Table 1**), our analysis of BF treated spikelets and composite APs equivalently.

Barrage-firing APs exhibited stereotyped changes in firing frequency after their induction. Following the induction of this form of firing in NG cells by a train of 2 ms-long current steps at 20 Hz, the average firing frequency of APs rose to a peak of 91.8 ± 11.1 Hz after 1.65 ± 0.24 s (*n* = 27) then gradually declined (**Figures 2A,B**; note the slightly lower peak frequency in this figure due to the different analysis method; Materials and Methods).

We performed similar experiments in FS cells in the piriform cortex, identified in GAD67-GFP mice by their GFP fluorescence, morphology and electrical properties (Suzuki and Bekkers, 2010a; **Figures 3A–C**). BF was also present but much less common in FS cells (23%, *n* = 22; *p* < 0.01 compared with NG cells, Fisher’s exact test; **Figure 1E**). BF in FS cells was similar to that in NG cells. For example, hyperpolarized spikelets were also seen in FS cells (**Table 1**), and the mean BF frequency also showed a peak following cessation of the triggering stimulus (although the frequency of APs during BF was generally lower than that of APs during the induction phase; **Figure 2C**). However, BF in FS cells required 2–3 times as many evoked APs for its induction, compared with NG cells, and its duration was about one third as long (**Table 1**). Thus, by several measures, BF is more reluctant in FS cells.

The frequency of occurrence of BF was as common in NG cells in the somatosensory cortex (83%; *n* = 6; layers 1–3) and in the CA1 region of the hippocampus (83%; *n* = 6; *Stratum lacunosum-moleculare*) as it was in NG cells in the piriform cortex (85%; *p* > 0.1, ANOVA; layers 1–3; **Figure 1E**; **Table 1**). However, BF was never seen in any other class of interneuron in the piriform cortex (*n* = 33) or in layer 2 principal cells (*n* = 20) (**Figure 1E**).

### BARRAGE FIRING CAN ALSO BE INDUCED IN NG CELLS *IN VIVO*

If BF is physiologically important, it should also occur *in vivo*. We tested this idea by using a two-photon microscope to make targeted whole-cell recordings from visualized NG cells in layers 1 and 2/3 of the somatosensory cortex of 30–45 d-old urethane-anesthetized mice (**Figure 4A**). Putative NG cells were initially identified by their very bright GFP fluorescence in GAD67-GFP mice (Suzuki and Bekkers, 2010a). After achieving a whole-cell recording from one of these cells, depolarizing current steps often revealed the delay to firing near rheobase that is characteristic of NG cells (**Figure 4B**; Suzuki and Bekkers, 2010a), although this behavior could be ambiguous in the presence of the prominent synaptic activity that is often seen *in vivo* (London et al., 2010). Finally, at the end of the experiment, an image stack of the cell was acquired to confirm that it had the fine-caliber, aspiny, multipolar dendritic arbor typical of NG cells (**Figure 4A**).

Using the 1 s step induction protocol, BF could be induced in a subset of NG cells *in vivo* (61%, *n* = 33; **Table 1**). This was a smaller percentage than for the corresponding cells *in vitro* (83%), and induction required about twice the number of APs (851 ± 96, *n* = 20; *cf.* 391 ± 70, *n* = 4; significantly different, *p* < 0.001). Otherwise, the properties of BF *in vivo* were similar to those *in vitro* (**Table 1**; **Figure 4C**), after allowing for the distorting effect of the higher electrode series resistance *in vivo* (Methods). For example, the AP threshold *in vivo* was significantly hyperpolarized in barrage-firing APs compared with regular APs (*p* < 0.001; **Table 1**), and barrage-firing AP frequency peaked soon after induction, just as was seen in slices (**Figure 2D**; **Table 1**). Furthermore, when a full-height barrage AP occurred *in vivo*, it always rode on top of a spikelet (**Figure 4C**, insets) and had a significantly slower rise time than control APs (*p* = 0.001, paired *t*-test; **Table 1**). These findings again implicate a distal axonal initiation site for BF *in vivo*.

Despite these similarities between slices and intact tissue, it appears that BF is less common and more difficult to trigger in NG cells *in vivo* (**Table 1**). A possible explanation is that *in vivo* experiments used older animals (40–45 d-old *cf*. 18-25 d-old for slices) and the ability to trigger BF might decline with age. This was examined by repeating the slice experiments with 40–45 d-old mice. The properties of BF were found to be similar in slices from younger and older mice (piriform NG cells from 40 to 45 d-old mice: 86% exhibited BF, 326 ± 34 APs before BF start [*p* = 0.04 compared with 18–25 d-old mice], BF duration, 48.4 ± 13.3 s [*p* = 0.6], *n* = 6 cells; neocortical NG cells from 40 to 45 d-old mice: 100% exhibited BF, 588 ± 26 APs before BF start [*p* = 0.06], BF duration, 30.2 ± 12.0 s [*p* = 0.98], *n* = 7 cells).

In summary, BF can be induced in cortical NG cells *in vivo* but is less commonly observed and more difficult to initiate than in slices, for reasons that are unclear. Nevertheless, its ability to be induced *in vivo* suggests that it may play an important physiological role.

### BARRAGE FIRING CAN BE TRIGGERED UNDER CONDITIONS OF HYPEREXCITABILITY

We hypothesized that NG cells might integrate recurrent excitation in the cortex, eventually causing these cells to fire persistently and produce lasting inhibition of overactive circuits. This idea is consistent with the report that GABA released by NG cells spills out of the synaptic cleft, providing diffuse, or tonic inhibition (Olah et al., 2009; Karayannis et al., 2010; Capogna and Pearce, 2011; Fishell and Rudy, 2011). We tested this hypothesis in two ways.

**FIGURE 5:**
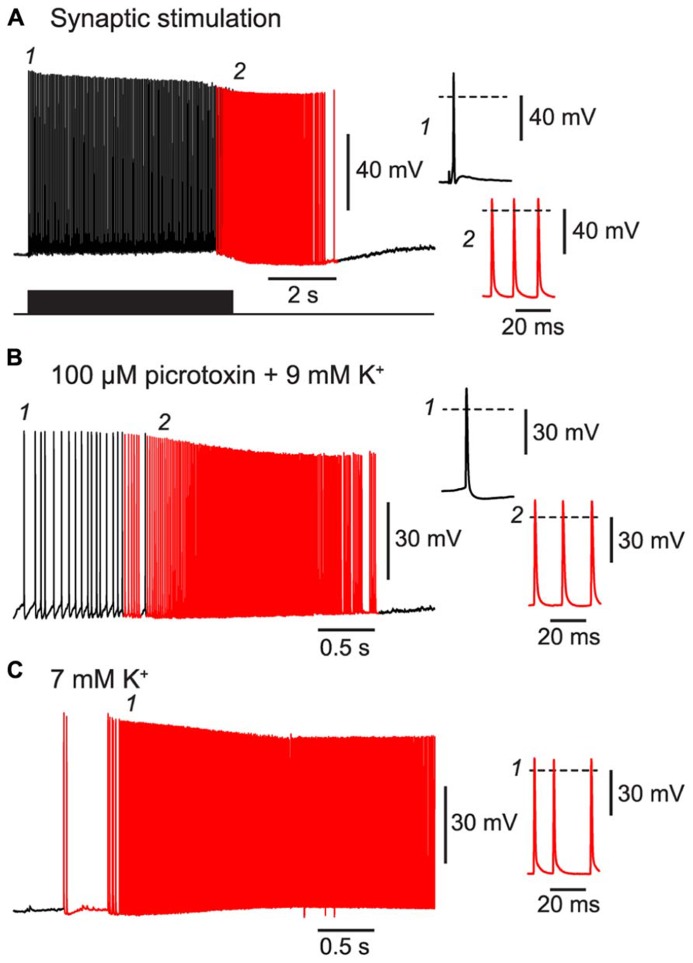
**BF can be triggered by trains of excitatory synaptic stimulation and by perfusing with proconvulsant bath solutions.** All panels show recordings from a NG cell in layer 3 in acute slices of the piriform cortex. **(A)**, A train of extracellular synaptic stimuli at 20 Hz induces BF. Bottom trace shows the timing of the stimuli; red portion of trace indicates barrage-firing APs. **(B)**, Perfusion with a modified ACSF containing 100 μM picrotoxin and 9 mM KCl triggers barrage-firing APs (red portions of trace), identified by their strongly hyperpolarized voltage thresholds. **(C)**, Perfusion with ACSF containing 7 mM KCl but no synaptic blockers also triggers barrage-firing APs. In this cell, low-threshold barrage-firing APs emerged without the prior activity of normal-threshold APs. In all panels, insets at right show (expanded) the AP responses at the time points indicated in the main panels at left (1, 2). Dashed lines indicate 0 mV.

First, we confirmed that BF could be triggered by patterns of activity resembling those that occur during seizures. For example, a 20 Hz train of excitatory synaptic stimulation was also effective at inducing BF in NG cells, like the direct current injection used above (**Figure 5A** *cf.* **Figure 1E**; 80% of synaptically excited cells exhibited BF; see **Table 1** for other parameters). Moreover, BF was readily triggered in NG cells in slices made hyperexcitable by blocking synaptic inhibition or raising the extracellular K^+^ concentration (manipulations commonly used in *in vitro* models of epilepsy; Jensen and Yaari, 1997; **Figures 5B,C**; 63% of NG cells treated in this way exhibited BF, *n* = 8 cells; see **Table 1** for other parameters). In some cases (*n* = 2), elevated K^+^ caused the emergence of APs without the prior occurrence of regular, depolarization-evoked APs (**Figure 5C**). Interestingly, isolated spikelets were not seen in any of the experiments using elevated K^+^, possibly because the K^+^ depolarization enabled spikelets to reliably trigger full-amplitude perisomatic spikes. In summary, these experiments indicate that NG cells can react to proconvulsant conditions by generating BF.

### BARRAGE-FIRING NG CELLS CAN SYNAPTICALLY INHIBIT NEARBY PRINCIPAL CELLS

**FIGURE 6:**
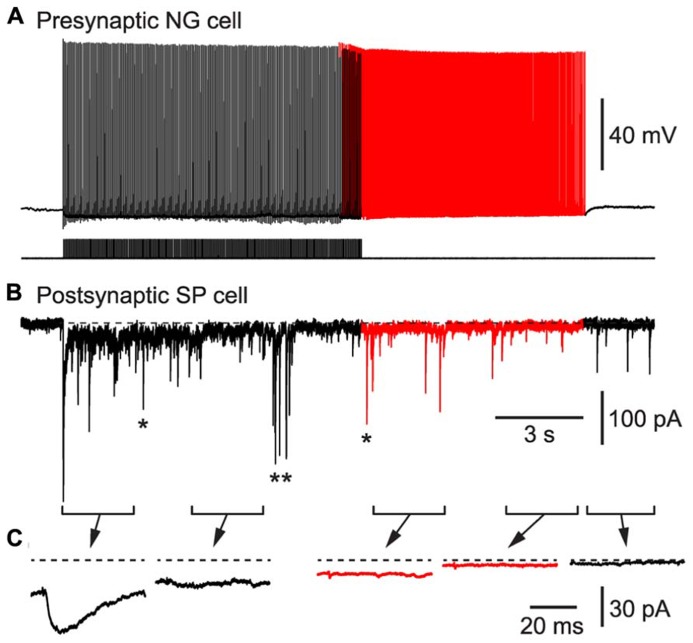
**BF in a NG cell produces persistent synaptic inhibition in a nearby superficial pyramidal (SP) cell in an acute slice of the piriform cortex.** **(A)**, Induction of BF (red portion of trace) by depolarizing current steps (20 Hz, 1.4 nA for 2 ms) in a layer 3 NG cell. **(B)**, IPSCs recorded in a SP cell that is postsynaptic to the NG cell in **(A)**. Red portion of trace corresponds to the period of BF in the presynaptic NG cell. In addition to the IPSCs generated by the presynaptic NG cell, isolated spontaneous IPSCs (e.g., *) or bursts of IPSCs (**) arising from other interneurons are also present. **(C)**, Spike-triggered averages of IPSCs recorded during each of the 2.5 s-long periods shown in **(B)**. Dashed horizontal line indicates the baseline prior to stimulation.

As a second test of our hypothesis, we determined whether BF APs in NG cells could produce synaptic inhibition in nearby glutamatergic neurons. We made pair recordings from a connected NG cell and a layer 2 principal neuron in the piriform cortex (**Figure 6**). Inhibitory postsynaptic currents (IPSCs) generated by NG cells are known to depress during trains (Karayannis et al., 2010). Hence, a spike-triggered average of IPSCs in the postsynaptic pyramidal cell declined over seconds until only a pedestal of synaptic current remained (**Figure 6C**). Nevertheless, when BF emerged (in this example, after ~10 s) APs were still capable of generating a small, plateau IPSC (7.8 ± 2.3 pA, or 4.7 ± 1.3% of the initial peak IPSC amplitude, *n* = 6 pairs). This IPSC was blocked by an inhibitor of GABA_A_ receptors (100 μM picrotoxin; not illustrated). Hence, BF in NG cells can drive lasting synaptic inhibition in nearby pyramidal cells.

## DISCUSSION

**FIGURE 7:**
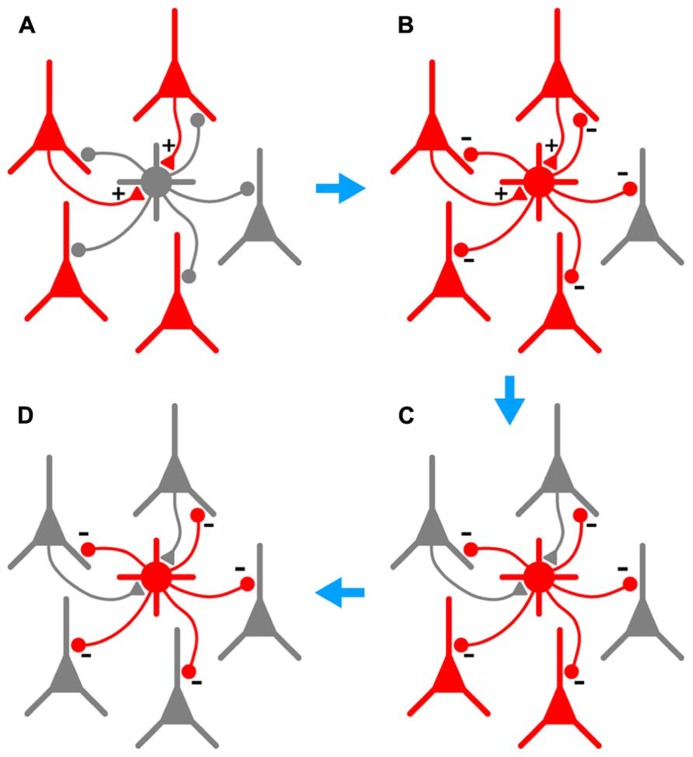
**Hypothesized series of steps by which BF could lead to autonomous and generalized inhibition in response to cortical hyperexcitability.** **(A)**, A population of pyramidal cells becomes hyperexcitable (red). A subset of these pyramidal cells synaptically excites a nearby NG or FS interneuron (+ symbols on gray cell at center). **(B)**, As a result of this excitation, the interneuron begins to fire repeatedly (gray cell becomes red), synaptically inhibiting a large number of pyramidal cells in its vicinity (- symbols). **(C)**, This inhibition eventually silences a subset of pyramidal cells, including those that are synaptically exciting the interneuron (upper two pyramidal cells become gray). However, BF ensures that the interneuron keeps firing after the presynaptic pyramidal cells fall silent, allowing the interneuron to continue inhibiting other nearby pyramidal cells that do not excite it. **(D)**, In due course all hyperexcitable pyramidal cells near the interneuron are silenced (all become gray). BF in the interneuron eventually ceases (not shown). Note that the scheme depicted here would only work in the cerebral cortex; hippocampal NG cells do not provide feedback inhibition to pyramidal cells (Price et al., 2005).

In this paper we examine further the phenomenon of persistent firing in GABAergic interneurons (Sheffield et al., 2011), more recently called retroaxonal barrage firing to distinguish it from other forms of persistent firing (Major and Tank, 2004; Sheffield et al., 2013; Thuault et al., 2013). We report that BF is most commonly expressed by NG cells in the cerebral cortex and hippocampus (>80% in piriform cortex, somatosensory cortex and hippocampus *in vitro*, ~60% in somatosensory cortex *in vivo*). A substantial minority of FS cells in the piriform cortex (~23%) also express this kind of firing, but it was never seen in any other class of interneurons or in principal neurons in the piriform cortex. We further show that BF can be induced in NG cells in the somatosensory cortex *in vivo*, although it appears to be more reluctant to initiate *in vivo* than *in vitro*. Our *in vivo* findings are, of course, subject to the usual provisos about the use of general anesthetics (Zhan and Luo, 2010). However, the induction of BF appears to be a local or cell-autonomous phenomenon (Sheffield et al., 2011, 2013) and may therefore be less susceptible to the subtle, global modulation of network processing thought to be produced by general anesthesia (Alkire et al., 2008; Brown et al., 2011). Finally, we report that BF is triggered by manipulations that replicate the excessive excitation that occurs during seizures, and that NG cells exhibiting this behavior can synaptically inhibit nearby pyramidal cells. This leads us to propose that NG cells (and perhaps also FS cells) are equipped to monitor excitability in their local neighborhood, providing a diffuse kind of inhibition that can outlast the original stimulus. That is, we suggest that barrage-firing interneurons can provide a generalized and autonomous form of inhibition that is triggered by over-excitation (see **Figure 7** and its legend for a fuller explanation of this idea).

Our findings are consistent with a recent report that BF is expressed by 82% of Ivy cells (a type of NG cell) and <20% of parvalbumin-positive fast-spiking cells in the mouse hippocampus (Krook-Magnuson et al., 2011). However, our work goes further by confirming that BF occurs with similar prevalence in NG cells in the archicortex (hippocampus), paleocortex (piriform cortex) and somatosensory neocortex across a range of ages (18–45 d-old animals). Importantly, we also show that BF can be induced in the cortex *in vivo*, supporting the view that it is not an artifact of slice recordings and may have evolved to perform physiologically important functions. Finally, we explore a variety of manipulations by which BF can be induced (synaptic stimulation, elevated K^+^, network disinhibition).

Exactly how BF is triggered is currently unknown, but a number of clues have emerged. The μ-opioid receptor agonist, DAMGO, has been reported to increase the number of APs required to trigger BF, possibly because of DAMGO-induced hyperpolarization (Krook-Magnuson et al., 2011). More recently it has been reported that BF may be triggered by a multicellular mechanism involving calcium signaling and gap junctions, possibly between non-neuronal cells (Sheffield et al., 2013). Our experiments do not directly address induction mechanisms, but some of our findings are illuminating. We find that BF is restricted to NG cells and FS cells in the piriform cortex, which are two cell classes that possess a dense axonal plexus (Suzuki and Bekkers, 2010a) and which also express profuse gap junctions among themselves and with other interneurons (Hestrin and Galarreta, 2005; Simon et al., 2005; Zsiros and Maccaferri, 2005; Fukuda et al., 2006). Thus, any mechanism for BF induction that involves gap junctions and/or ectopic interactions between dense axonal arbors would be plausible.

In addition to their tendency to fire persistently, NG cells have other properties that might suit them to providing protection against seizures. NG-like interneurons are found in many brain regions (Capogna and Pearce, 2011; Fishell and Rudy, 2011) and, at least in the piriform cortex, are distributed across all laminae (Suzuki and Bekkers, 2010a,b). Strong gap junction coupling between NG cells can help to enforce synchronized firing of many NG cells in a network (Simon et al., 2005; Zsiros and Maccaferri, 2005; Olah et al., 2007). Hence, NG cells could provide uniform protective coverage over wide regions of the cortex. NG cells also produce spillover or volume transmission (Capogna and Pearce, 2011) which potentially enables them to inhibit a large number of neuronal membranes in their vicinity, or to activate presynaptic GABA_B_ receptors, suppressing transmitter release. BF and the resultant release of GABA from NG cells might enhance tonic inhibition via extrasynaptic GABA_A_ receptors (Capogna and Pearce, 2011). Low levels of persistent inhibition might also alter the intrinsic excitability of pyramidal cells by changing their input-output gain (Mitchell and Silver, 2003). However, because GABAergic transmission from NG cells readily depresses with repeated stimulation (Karayannis et al., 2010; **Figure 6**), BF-induced inhibition provided by these cells will be self-limiting. The inhibitory transmission provided by FS neurons does not depress so profoundly in trains (Suzuki and Bekkers, 2010a) and so, despite their lesser expression of BF (23% of FS cells *cf*. ~60–85% of NG cells; Results), FS cells might be functionally more important in providing persistent inhibition in response to seizures. Of course, postsynaptic factors, like desensitization of GABA_A_ receptors, might also set limits on the effectiveness of persistent GABA release (Jones and Westbrook, 1996).

Although this paper focuses on the hypothesis that BF serves to suppress epileptic activity, other possible functions of BF should not be ruled out (Sheffield et al., 2011). BF might be involved in the generation of oscillations in electrical activity that are commonly observed in the cortex, including the piriform cortex (Kay et al., 2009). Alternatively, BF might be important for storing information for short periods of time, as for working memory (Thuault et al., 2013), or it might enable a novel form of communication between neurons and glia (Sheffield et al., 2013).

Assuming that BF does play a role in seizure suppression, what might be its significance for an understanding of epilepsy? The factors determining the onset of epileptic seizures have been intensively studied and are known to be very variable, reflecting the diversity of classes of epilepsies (Harlen, 1997). In contrast, the factors that determine seizure termination have been less studied. Partly this reflects the complexity of the ictal state, during which many neuronal parameters are far from equilibrium across wide areas of the brain. A number of factors have been suggested to be important for termination, including generalized alterations in transmembrane ion gradients, changes in levels of neuromodulators and metabolites, and activation or inactivation of ion channels (see Lado and Moshe, 2008, for review). Here we propose a different idea, namely, that there are specialized inhibitory circuits with the purpose of providing a kind of global feedback inhibition in response to over-excitation; i.e., their role is to be a “safety valve” for the brain. Future work would need to test this idea using more realistic *in vivo* models of epilepsy, and to establish whether there is, indeed, a direct causal link between BF and the termination of seizures.

## Conflict of Interest Statement

The authors declare that the research was conducted in the absence of any commercial or financial relationships that could be construed as a potential conflict of interest.
